# Ticks as potential vectors of *Mycobacterium leprae*: Use of tick cell lines to culture the bacilli and generate transgenic strains

**DOI:** 10.1371/journal.pntd.0007001

**Published:** 2018-12-19

**Authors:** Jéssica da Silva Ferreira, Diego Augusto Souza Oliveira, João Pedro Santos, Carla Carolina Dias Uzedo Ribeiro, Bruna A. Baêta, Rafaella Câmara Teixeira, Arthur da Silva Neumann, Patricia Sammarco Rosa, Maria Cristina Vidal Pessolani, Milton Ozório Moraes, Gervásio Henrique Bechara, Pedro L. de Oliveira, Marcos Henrique Ferreira Sorgine, Philip Noel Suffys, Amanda Nogueira Brum Fontes, Lesley Bell-Sakyi, Adivaldo H. Fonseca, Flavio Alves Lara

**Affiliations:** 1 Lab. de Microbiologia Celular, Oswaldo Cruz Institute, Oswaldo Cruz Foundation, Rio de janeiro, Brazil; 2 Department of Animal Parasitology, Institute of Veterinary Medicine, Federal Rural University of Rio de Janeiro, Rio de janeiro, Brazil; 3 Lauro de Sousa Lima Institute, Department of Biology, Bauru, Brazil; 4 Lab. de Hanseníase, Oswaldo Cruz Institute, Oswaldo Cruz Foundation, Rio de janeiro, Brazil; 5 School of Agricultural Sciences and Veterinary Medicine, Pontifical Catholic University of Parana, Curitiba, Brazil; 6 Lab. de Bioquímica de Artrópodes Hematófagos, Institute of Medical Biochemistry Leopoldo de Meis, Federal University of Rio de Janeiro, Rio de Janeiro, Brazil; 7 Lab. de Biologia Molecular Aplicada a Micobactérias, Oswaldo Cruz Institute, Oswaldo Cruz Foundation, Rio de Janeiro, Brazil; 8 Department of Infection Biology, Institute of Infection and Global Health, University of Liverpool, Liverpool, United Kingdom; Yale University, UNITED STATES

## Abstract

Leprosy is an infectious disease caused by *Mycobacterium leprae* and frequently resulting in irreversible deformities and disabilities. Ticks play an important role in infectious disease transmission due to their low host specificity, worldwide distribution, and the biological ability to support transovarial transmission of a wide spectrum of pathogens, including viruses, bacteria and protozoa. To investigate a possible role for ticks as vectors of leprosy, we assessed transovarial transmission of *M*. *leprae* in artificially-fed adult female *Amblyomma sculptum* ticks, and infection and growth of *M*. *leprae* in tick cell lines. Our results revealed *M*. *leprae* RNA and antigens persisting in the midgut and present in the ovaries of adult female *A*. *sculptum* at least 2 days after oral infection, and present in their progeny (eggs and larvae), which demonstrates the occurrence of transovarial transmission of this pathogen. Infected tick larvae were able to inoculate viable bacilli during blood-feeding on a rabbit. Moreover, following inoculation with *M*. *leprae*, the *Ixodes scapularis* embryo-derived tick cell line IDE8 supported a detectable increase in the number of bacilli for at least 20 days, presenting a doubling time of approximately 12 days. As far as we know, this is the first *in vitro* cellular system able to promote growth of *M*. *leprae*. Finally, we successfully transformed a clinical *M*. *leprae* isolate by inserting the reporter plasmid pCHERRY3; transformed bacteria infected and grew in IDE8 cells over a 2-month period. Taken together, our data not only support the hypothesis that ticks may have the potential to act as a reservoir and/or vector of leprosy, but also suggest the feasibility of technological development of tick cell lines as a tool for large-scale production of *M*. *leprae* bacteria, as well as describing for the first time a method for their transformation.

## Author summary

Leprosy is a slow-progressing and extremely debilitating disease; the armadillo is the only animal model able to mimic the symptoms observed in humans. In addition, the causative agent, *Mycobacterium leprae*, is not cultivable *in vitro*. Due to these constraints the chain of transmission is still not yet completely understood. We know, however, that at least two animals, armadillos in the Americas and red squirrels in the UK, are natural reservoirs of the bacillus, although their role in disease epidemiology is unclear. This information raised the following question: Can ticks carry leprosy from wild animals to humans? In the present study we demonstrated that artificially-infected female cayenne ticks are able to transmit the bacillus to their offspring, which were then able to transmit it to rabbits during bloodfeeding. We were able to grow *M*. *leprae in vitro* in a tick cell line for the first time. We also generated the first transgenic *M*. *leprae* strain, making the pathogen fluorescent in order to monitor its viability in real time. We believe that this new methodology will boost the screening of new drugs useful for control of leprosy, as well as improving understanding of how *M*. *leprae* causes disease.

## Introduction

Leprosy, also known as Hansen’s disease, is a chronic infectious disease caused by the intracellular pathogen *Mycobacterium leprae*, manifested mainly as dermatoneurological signs and symptoms with high potential to progress causing physical disabilities and deformities.

Although it is currently agreed that leprosy is transmitted through the prolonged contact of genetically susceptible individuals with untreated multibacillary patients, the epidemiology of leprosy displays some features that are not well explained by this sole mode of transmission, particularly its geographic distribution. The disease, which during human history has spread throughout the globe [1], was eradicated in Europe almost a century before rifampicin discovery [2], and is now found mainly in tropical regions [3].

Several studies address the impact of environmental factors in the endemicity of leprosy, including factors such as poverty [4], human migration [5], population density [6], contact with armadillos [7] and environmental humidity [8]. One of the factors which is most strongly associated with the disease in Brazil is deforestation for agricultural activities [9]. In Brazil, new cases of leprosy are mainly concentrated in populations living on the agricultural borders, in the north and -west-central parts of the country, with 3.58 new cases / 10,000 inhabitants, contributing 68.18% of the new cases registered countrywide in 2015. These populations, who live in the countryside, are apparently more exposed to leprosy than the inhabitants of the great cities of the southeast region, where despite higher population density and public health vigilance, only 14.05% of new cases were notified, according to the Health Hazard Notification System (SINAN) of the Brazilian Ministry of Health. Although leprosy is not currently recognized as a zoonosis outside the southern USA [10] and the northern region of Brazil [11], several studies point to the potential of the armadillo *Dasypus novemcinctus* in other parts of the Americas [10], non-human primates around the world [12,13], and more recently red squirrels in the United Kingdom [14] as potential reservoirs of *M*. *leprae*. Some of these animals are frequently infested by ticks. Since the beginning of the last century, studies have been performed in an attempt to implicate the action of hematophagous arthropods in the transmission of leprosy [15]. Although efforts have been made in describing the supposed role of mosquitoes, such as *Aedes aegypti* [16–18] and *Culex fatigans* [19], and flies [20] in leprosy transmission, these studies were based only on microscopic observations of acid-fast bacilli inside the insects’ digestive tracts, without concurrent *M*. *leprae* identification by molecular tools.

More recently, we have presented experimental evidence indicating that triatomine bugs, already associated with the transmission of another mycobacterial disease, Buruli ulcer [21], have potential as vectors of leprosy, being able to excrete infective bacilli in their feces at least 20 days after the blood meal [22]. The first report of the possibility of ticks acting as vectors of leprosy dates back to the 1940s, when acid-fast bacilli were observed in intestinal macerates of ticks of the genus *Amblyomma* after blood feeding on a skin lesion of a boy afflicted by the disease [23].

The three-host tick *Amblyomma sculptum* is a member of the *Amblyomma cajennense* sensu lato complex [24]; *A*. *cajennense* s.l. is distributed throughout tropical and sub-tropical areas of the American continent, from the southern US and Central America to northern Argentina, with the exception of Chile, Uruguay and the extreme south of Brazil, following the current distribution of leprosy in the Americas [25]. Humans are most commonly attacked by the immature stages (larvae and nymphs) of *A*. *sculptum*, while adults prefer to parasitize other mammals such as horses, cattle, tapirs, capybaras, domestic and wild canids, as well as birds, armadillos and rodents [26]. This tick species is implicated in the transmission of Rocky Mountain spotted fever to humans [26,27], and has been shown to have the ability to transfer pathogens such as *Rickettsia rickettsii* to its eggs [28]. Because of its wide geographic distribution and important role as a vector of human pathogens in South America, *A*. *sculptum* was chosen as a model for the present study.

Tick cell lines have often been used for the isolation and growth of fastidious or previously non-cultivable bacterial pathogens transmitted by ticks and other arthropods [29–33]. These lines exhibit some advantageous characteristics which could be critical for the *in vitro* cultivation of *M*. *leprae*, such as the ability to survive for long periods, measured in months or even years, in the same culture container requiring only regular changes of medium [34]. They also grow relatively slowly, at temperatures between 28°C and 34°C [30], the same range required by *M*. *leprae* for survival [35].

Here we provide evidence for the ability of *A*. *sculptum* to acquire and transmit, under controlled experimental conditions, the *M*. *leprae* bacillus during blood feeding. In addition, we describe a protocol for the *in vitro* production of *M*. *leprae* in the *Ixodes scapularis* tick cell line IDE8. Once we were able to grow this mycobacterium *in vitro*, we generated the first transgenic *M*. *leprae* strain to be described, through transfection with the reporter plasmid pCHERRY3. We believe that this methodology will change the paradigm of studies on leprosy, making possible continuous *in vitro* cultivation and generation of mutant strains of *M*. *leprae*, thereby enabling rapid progress in many areas including identification of virulence factors and drug discovery.

## Materials and methods

### *Amblyomma sculptum* source and maintenance

*A*. *sculptum* ticks from the Lab. de Doenças Parasitárias colony at the Federal Rural University of Rio de Janeiro (UFRRJ) were maintained on male rabbits (*Orictolagus cuniculus*, aged between 60 and 90 days and weighing approximately 2 Kg, provided by Veterinary Institute Cuniculture, UFRRJ), by a modification of a previously-described method [36]. A small area of the dorsal region was shaved, and bags of cotton cloth were glued to the rabbit’s skin. Within these bags were placed 20 pairs of adult male and female *A*. *sculptum*. To prevent removal of the bags by the rabbits, plastic cervical collars were placed on the necks of the animals as previously described [37].

### *Mycobacterium leprae* preparation

*Mycobacterium leprae* Thai-53 strain [38], originally donated by Dr. James Krahenbuhl (National Hansen’s Disease Program, Laboratory Research Branch, Louisiana State University, Baton Rouge, LA, USA) was used to infect athymic nude mice bred in the Lauro de Souza Lima Institute (Baurú, São Paulo, Brazil) under germ-free conditions with 10^4^ bacilli in each posterior footpad. After six months the mice were sacrificed by a lethal dose of ketamine (300 mg/Kg) associated with xylazine (30 mg/Kg) (Syntec, São Paulo, Brazil), skin and bones were removed from the footpads aseptically in RPMI medium, and the remaining tissues were cut into small pieces with sterilized scissors and tweezers. The tissues were then gently dissociated for 2 h at 33°C in a solution comprising 15 mg/mL collagenase type I, 7 mg/mL dispase, 50 μg/mL DNAse and 100 μg/mL ampicillin (Life Technologies, Carlsbad, CA, USA) dissolved in Milli-Q (Millipore, Darmstadt, Germany) ultrapure water. After digestion, the resultant suspension was transferred to 1.5 mL tubes, centrifuged at 12,000 x g for 5 min and the supernatant was discarded. The pellet was washed twice with 1.5 mL water at 12,000 x g for 5 min, resuspended in 0.1 N sodium hydroxide (Sigma, St. Louis, MI, USA) and immediately centrifuged at 12,000 x g for 5 min. The purified bacilli were washed twice with 1.5 mL RPMI medium (LGC, Cotia, SP, Brazil) and the pellet was mechanically dissociated by passing the suspension through a 26 gauge needle. *M*. *leprae* preparations supplemented with 10% fetal bovine serum (FBS) (Cripion, Andradina, SP, Brazil) were stored for up to 3 days at 4°C. For the transformation protocol, *M*. *leprae* isolated from a skin lesion of a multibacillary leprosy patient from Igarapé Açú, Pará, northern Brazil, was used. This strain, designated PA4, had been cultivated in nude mouse footpads for six months and genotyped as a 4N strain.

The viability of both *M*. *leprae* strains was determined by assessing membrane integrity using the LIVE/DEAD BacLight bacterial viability kit (Life Technologies, CA, USA), performed according to the manufacturer’s instructions [39]. The percentage of viable bacilli in the preparation were estimated by counting the numbers of living bacteria (Cyto9 green signal excited at 488nm) and dead bacteria (propidium iodide red signal excited at 470nm), using an Axio Observer Z1 Zeiss microscope, with a HXP light source. Twenty fields each presenting at least 50 bacteria were quantified per preparation. Only bacterial preparations with viability of at least 85% as determined by the LIVE/DEAD method were used in the present work. Sterility of *M*. *leprae* preparations was checked by inoculation onto BHI and 7H9 liquid media (BioCen, Campinas, SP, Brazil), and Lowenstein and blood agar plates (BioCen, Campinas, SP, Brazil), which were monitored daily for 72 h (BHI and blood agar) and 45 days (7H9 and Lowenstein) at 37°C in order to exclude the presence of other contaminating microorganisms, including mycobacteria.

### Artificial tick feeding and infection

Nearly-engorged adult female *A*. *sculptum* were detached from rabbits and weighed before being artificially fed, using plastic pipette tips [40,41] as follows. The ticks were divided into two groups; one group received at least 400μL of citrated rabbit blood containing 10^7^/mL viable *M*. *leprae* Thai-53 strain, and the other group received the same volume of uninfected blood. The tick mouthparts were introduced into the tips at an angle of approximately 30° and the ticks were maintained at 37±1°C with relative humidity of 80% for 24 h, when they were weighed again and ticks that had not gained at least 0.2 g were discarded. Engorged ticks were maintained at 80% humidity and 27°C for between 2 h and 15 days prior to dissection or for oviposition. Artificially-fed ticks were dissected and tissue samples were collected individually and stored in TRIzol (Thermo Fisher Scientific, MA, USA) or 10% buffered formalin; midguts were collected after 2 h and 15 days, while ovaries were collected after 2 days. Pools of 0.2 g eggs laid by the artificially-fed ticks were collected on the second and third days after the blood meal and homogenized in TRIzol. Larvae were obtained from the same amounts of eggs after 30 days’ incubation in sterile syringes [42] at 27°C with relative humidity approximately 80%. Two days after hatching, larvae were homogenized in TRIzol to investigate presence of *M*. *leprae* DNA and RNA.

### Analysis of the potential of blood feeding larval ticks to transmit *M*. *leprae*

Two days after hatching, larvae originating from 0.2g of eggs laid by artificially-infected female *A*. *sculptum* were fed on a rabbit as described above for five days, the time required for this developmental stage to complete engorgement, and then removed. Subsequently, biopsies (5 mm punch) were performed in areas of the rabbit’s skin where there was evidence of previous insertion of the larval mouthparts. The biopsies were divided into two parts using a sterile scalpel; one part was extracted in TRIzol for quantitative PCR (qPCR) analysis of bacillary viability as described below, and the other part was fixed in 10% buffered formalin to perform immunolocalization of *M*. *leprae* as described below.

### Analysis of *M*. *leprae* viability by qPCR

RNA and DNA were simultaneously extracted from test and control samples using TRIzol through a modification of the previously-described single-tube RNA extraction protocol [43]. Briefly, TRIzol was added to all samples to give a total volume of 1 ml. This solution was transferred to FastRNA tubes (FastRNA kit-Blue; MP Biomedicals, Santa Ana, CA). Samples were homogenized twice in the FastPrep FP 24 instrument (MP Biomedicals) at a speed setting of 6.5 for 45 s. Tubes were allowed to cool for 2 min between each round of homogenization. After homogenization, tubes were chilled on ice for 5 min, 200 μl chloroform-isoamyl alcohol (24:1) was added, and tubes were vortexed for 10 s and then centrifuged at 700 × *g* at 4°C for 5 min. The supernatant was transferred to another tube and centrifuged again at 12,000 × *g* for 10 min at 4°C. RNA was purified from 400 μl of the aqueous phase, any contaminating DNA was removed from the RNA preparations using the DNA-free kit (Ambion, Inc., Austin, TX) following the manufacturer’s instructions, the RNA was precipitated with isopropanol, washed with 70% ethanol, finally resuspended in 30 μl diethyl pyrocarbonate-treated H_2_O, and stored at -80°C until use [44]. DNA was purified from the remaining aqueous phase and interphase of the FastRNA tubes. Briefly, 100 μl of 10 mMTris-EDTA (pH 8.0) and 150 μl chloroform-isoamyl alcohol (24:1) were added to the remaining aqueous phase and interphase (500 μl) and homogenized in the FastPrep FP 24 instrument twice. After centrifugation at 12,000 × *g* for 10 min, the aqueous phase was transferred into another tube and precipitated with 0.1 volume of 3 M sodium acetate pH 5.2 and two volumes of cold ethanol. The DNA pellet was washed in 70% ethanol, dissolved in 30 μl of sterile distilled water, and stored at -80°C until use. Qualitative RNA analysis was performed by agarose gel electrophoresis and quantification of both DNA and RNA was performed in a NanoDrop One apparatus (Thermo Fisher Scientific, MA, USA). The RNA was reverse-transcribed using random primers and Superscript III (Invitrogen, CA, USA) following the manufacturer’s instructions.

The levels of *M*. *leprae* 16S rRNA (as inferred from the resultant cDNA) and genomic DNA were determined in all tissues by qPCR, using as primer pair: sense 5’ GCA TGT CTT GTG GTG GAA AGC ‘3 and anti-sense 5’ CAC CCC ACC AAC AAG CTG AT ‘3, as described elsewhere [44]. The PCR protocol used was 50°C for 2 min and 95°C for 10 min, followed by 40 cycles of 95°C for 15 seconds and 60°C for 1 min, monitoring SYBR Green fluorescence in an ABI StepOne Plus System (Applied Biosystems, CA, USA). Viability of *M*. *leprae* was determined by calculating the qPCR 16S cDNA:DNA ratio, according to the formula 2^-(ΔCT)^, where ΔCT = *M*. *leprae* 16S cDNA CT–*M*. *leprae* 16S DNA CT [44,45]. In order to convert 2^-(ΔCT)^ qPCR signals into numbers of live *M*. *leprae* genomes, we generated standard curves by adding different concentrations of living *M*. *leprae*, ranging from 10^8^ to 10^3^, into uninfected rabbit skin biopsies, tick cell cultures and tissues: midguts dissected 2h and 15 days after the blood meal, pools of 0.2 g eggs harvested between 48h and 72h after the blood meal, and larvae originating from 0.2g of eggs harvested 48h after hatching. We did not include ovarian tissue in this analysis due to difficulties in assuring absence of contamination from midgut contents during dissection. The angular coefficient of each of the five standard curves was used to determine the number of viable *M*. *leprae* in each condition. Control tissues and samples without the addition of DNA were negative for both cDNA and DNA targets.

### Immunolocalization of *M*. *leprae* by fluorescence microscopy

As tick tissues are not well preserved by freezing protocols, *M*. *leprae* immunolocalization in tick tissues and rabbit skin biopsies was performed by fixation in 10% buffered formalin followed by standard paraffin embedding and sectioning as previously described [46]. The antigenic recovery was performed as follows: paraffin sections were dewaxed in xylol, hydrated in graded ethanol and distilled water, and heated in 10 mM sodium citrate buffer (pH 6.0) for 10 min in a 700 W microwave oven followed by 30 min to cool down to room temperature. *M*. *leprae* immunolocalization was performed using the monoclonal anti-lipoarabinomannan (LAM) antibody CS-35 reactive with *Mycobacterium* spp., kindly provided by the Biodefense and Emerging Infections Research Resources Repository: http://www.beiresources.org/TBVTRMResearchMaterials/tabid/1431/Default.aspx. Briefly, slides were permeabilized and blocked by 30 min incubation with 0.01% Triton X-100 and 10% FBS (Gibco/Life Technologies) in PBS pH 7.2 (permeabilization buffer). Then tissues were incubated for 2 h with the mouse IgG anti-LAM antibody CS-35 (diluted 1:50 vol/vol in permeabilization buffer) and nuclei were stained with 4',6-diamidino-2-phenylindole (DAPI Sigma-Aldrich, St. Louis, USA). Secondary antibodies, IgG anti-mouse conjugated with Alexa 594 (Invitrogen, CA, USA) diluted 1:250 in permeabilization buffer, were incubated with the samples for an additional 2 h and the tissues were then mounted in Vectashield (Vector Laboratories, CA, USA) and observed using a Zeiss Axio Observer Z1 microscope with a Colibri illumination system using a 590 nm LED with a Zeiss fluorescence filter 50 (Carl Zeiss, Heidenheim, Germany).

### *M*. *leprae* infection of tick and human cell lines

Tick cell lines AVL/CTVM17 derived from larval *Amblyomma variegatum* [47], HAE/CTVM8 derived from embryonic *Hyalomma anatolicum* [48] and IDE8 derived from embryonic *Ixodes scapularis* [49] were maintained at Lab. de Doenças Parasitárias, Institute of Veterinary Medicine (UFRRJ) as described previously [50]. All cell lines were seeded in 24-well culture plates at a density of 2x10^5^ cells / well in 1 mL of complete culture medium as follows. HAE/CTVM8 cells were cultivated in L-15/H-Lac medium comprising equal volumes of Leibovitz’ L-15 medium (Gibco/Life Technologies) supplemented with 20% FBS (Cripion, Andradina, SP, Brazil), 10% tryptose phosphate broth (TPB, Sigma-Aldrich, Missouri, USA) and 2mM L-glutamine (Sigma-Aldrich, Missouri, USA) (L-15), and Hanks’ balanced salt solution (HBSS, Sigma-Aldrich, Missouri, USA) supplemented with 20% FBS, 0.5% lactalbumin hydrolysate (Sigma-Aldrich, Missouri, USA) and 2mM L-glutamine (H-Lac). IDE8 cells were grown in L-15B medium [51] supplemented with 10% FBS, 10% TPB, 0.1% bovine lipoprotein-cholesterol concentrate (MP Biomedicals, UK) and 2 mM L-glutamine (L-15B). AVL/CTVM17 cells were cultivated in medium comprising equal volumes of L-15, H-Lac and L-15B. Cells destined for microscopical analysis were seeded on coverslips, and all tick cells were maintained at 30°C. Human monocyte THP-1 cells were obtained from the American Type Culture Collection and maintained in RPMI 1640 medium (LGC Bioscience, Cotia, SP) supplemented with 10% FBS (Cultilab, Campinas, SP), without antibiotics. THP-1 cultures were grown at 37°C and transferred to 30°C when infected, within a humidified 5% CO_2_ atmosphere. Cells were subjected to infection with *M*. *leprae* Thai-53 strain at a multiplicity of infection of 50 bacteria per cell (MOI 50:1) at 30°C. To examine association of *M*. *leprae* with tick cells, a total of 10^7^ freshly harvested *M*. *leprae* were stained with PKH67 dye (Sigma-Aldrich, Missouri, USA) following the manufacturer’s instructions with minor adaptations. Briefly 100μL of diluent provided with the kit and 1μL of PKH67 were added to the bacterial suspension. After 10 min incubation at room temperature with periodic mixing, 1mL of FBS was added to the bacterial suspension for 1 min to stop the reaction. The suspension was centrifuged at 14,000 x g for 5 min and the bacteria were resuspended in 100 μL of complete L15-B medium and immediately added to cell cultures seeded as above. For microscopy, after 24 h incubation at 30°C, cells in each well were fixed with 500 μL of 4% paraformaldehyde for 20 min, followed by three washes with HBSS and nuclear staining with DAPI. The coverslips were removed from the wells and placed on slides for analysis with a Zeiss Axio Observer Z1 fluorescence microscope as above. Samples were excited using 360 nm and 470 nm LEDs for respectively DAPI and PKH67, together with a Zeiss fluorescence filter 61. Images were acquired with a HMR Axiocam monochromatic camera controlled by Axiovision software version 3.2 (Carl Zeiss, Heidenheim, Germany), in which colors were arbitrarily attributed to the different channels. For flow cytometry, cells were gently washed with HBSS in order to remove any free bacilli, detached and fixed by the addition of 1% paraformaldehyde at 4°C until analysis using a BD Accuri C6 cytometer (BD Biosciences, California, USA) with the FL1-A channel set for counting 10,000 events. In both techniques, microscopy and flow cytometry, the index of bacterial association was expressed as the percentage of fluorescent cells.

In order to determine *M*. *leprae* growth inside tick cells, 2x10^5^ cells of each of the three cell lines were seeded in wells of a 24-well cell culture plate and infected 24 h later with live *M*. *leprae* Thai-53 strain, at a MOI of 50:1. After 24 h, 10 days and 20 days of infection, cultures were washed twice with sterile PBS and the cells were resuspended. The cell suspensions were centrifuged at 12,000 × g for 5 min and resuspended in milk-formaldehyde, which was prepared by centrifuging 1.5 mL of skimmed milk (Nestlé, Vaud, Switzerland) for 15 min at 12,000 × g, and transferring 1 mL of the supernatant to another tube containing 150 μL of formaldehyde (Sigma-Aldrich, Missouri, USA) and 9 mL of ultra-pure water. The pellets were resuspended at a dilution of 1:10 v/v in milk-formaldehyde and 10 μL aliquots were each spread over a 10 mm diameter circle etched on a glass slide (Electron Microscopy Sciences, Hatfield, USA). The glass slides were stained using the Kinyoun method for microscopic analysis [52]. Twenty fields with at least 50 bacteria were quantified per cell culture. The number of bacilli was estimated based on the ratio of the area of the circle on the slide area to the area of the field of view of the microscope obtained with a Zeiss Objective Plan-Apochromat microscope 100x/1.40 Oil objective (Carl Zeiss, Heidenheim, Germany). To calculate this ratio, the radius (r) of each area was measured using a 1mm microscope micrometer calibration slide ruler with 100 divisions (Cnscope, Beijing, China), and the areas were calculated using the formula π x r^2^. Thus, the area of the circle on the slide and the area of the microscope field were determined as 78,539 mm^2^ and 38 mm^2^ respectively. This ratio (2.07x10^3^) was employed in the following equation:

$$Number\;of\;bacilli\;per\;ml\;of\;cell\;culture=A\;x\;2.07x10^{3}\;x\;B\;x\;100$$

when A = average number of bacilli in ten microscope fields, B = dilution correction and 100 = volume correction from 10 μL to 1 mL.

*M*. *leprae* viability inside cells was determined by qPCR using 16S rRNA as target as described above. Briefly, 2x10^5^ cells of each of the three tick cell lines and the human monocyte THP-1 cells were seeded in 24-well cell culture plates. The differentiation of monocytes to macrophages was achieved by adding 200 ng/ml PMA (Sigma-Aldrich, Missouri, USA) to the medium. After 24 h, undifferentiated monocytes and dead cells were washed out with PBS, medium was resplaced, and another 24 h incubation preceded the infection with live *M*. *leprae* Thai-53 strain at a MOI of 50:1. After 20 days of infection, cultures were washed twice with sterile PBS and intracellular *M*. *leprae* DNA and RNA were extracted by adding TRIzol reagent.

### Shepard`s model test of viability of tick cell-derived *M*. *leprae*

An IDE8 cell culture was infected with *M*. *leprae* Thai-53 strain at a MOI of 50:1 at 30°C. Culture medium was changed by removal and replacement of two-thirds of the total volume at weekly intervals. After 20 days of incubation, cells were washed and homogenized in 0.05% Tween 80 in sterile water. *M*. *leprae* was pelleted by centrifugation at 12,000 x g for 10 min, resuspended in 100 μL of PBS and counted as described above. Fifteen BALB/c mice were divided into three groups of 5 animals. Two groups were inoculated with 20 μL per foot pad of *M*. *leprae* suspension containing 10^4^ bacilli harvested from the IDE8 culture in a conventional Shepard's infection model [53]. A negative control was generated by treating animals in one of the two groups that received IDE8-derived *M*. *leprae* with 10mg/kg/week rifampicin by gavage. As a positive control, the third group were inoculated with the same number of *M*. *leprae* freshly purified from nude mouse foot pads. After six months, the mice were sacrificed by an intraperitoneal overdose of ketamine (300mg/kg) and xylazine (30mg/kg) and their foot pads were excised and macerated for bacillary counting after Kinyoun staining as above.

### *M*. *leprae* transformation

An aliquot of 10^9^ bacteria of the *M*. *leprae* strain PA4 was subjected to transformation by electroporation with 5μg of pCHERRY3 plasmid DNA, kindly provided by Dr Suzie Hingley-Wilson of the University of Surrey, UK. This non-integrative plasmid, with a size of 6.3 kb, harbors a hygromycin resistance gene, a mycobacterial replication cassette, and the far-red fluorescence gene *mCherry* with expression driven by the *Mycobacterium smegmatis* promoter *Psmyc* [54]. The BioRad Micropulser TM (Hercules, CA, USA) electroporator parameters were adjusted for tension of 2.5 kV and electric resistance of 1000 Ω. The bacilli were pelleted by centrifuging for 10 min at 12,000 × g. The supernatant was discarded, and the pellet was dissolved in 200 μL sterile MilliQ water with 0.05% Tween 80. After adding the plasmid, the mixture was homogenized and transferred to a sterile electroporation cuvette at room temperature. After applying the electric pulse, transformed *M*. *leprae* were recovered with a sterile Pasteur pipette and immediately transferred to a 25 cm^2^ culture flask containing 10^7^ IDE8 cells, maintained with weekly medium changes as above. After one week, selection of transformed *M*. *leprae* was performed by adding 50 μg/mL of hygromycin. After 1 week, 2 weeks and 2 months, a small number of cells were gently detached from the culture flask by shaking, and 10^5^ cells were placed in a glass bottomed 1.9 cm^2^ cell culture dish (CELLview, Greiner Bio-One). mCherry expression by *M*. *leprae* was monitored using the Zeiss Axio Observer Z1 microscope as above, using a 590 nm LED with a Zeiss fluorescence filter 61 (Carl Zeiss, Heidenheim, Germany). Time-lapse photographic analysis of *M*. *leprae*-mCherry was performed 2 weeks after transformation, using 10^5^ cells placed in a glass bottomed CELLview culture dish and the same microscope with the Zeiss Definitive Focus (Carl Zeiss, Heidenheim, Germany) accessory with stage temperature stabilized at 30°C. The medium was supplemented with 150 μg / mL ofloxacin (Sigma, St. Louis, MI, USA), and 5 h later the video was started, with acquisition of one frame every 5 min over a period of 3 h.

### Genotyping by multiple Locus Variable number analysis (MVLA)

DNA extracts from *M*. *leprae*-infected tick cell cultures were submitted to genotyping by MLVA analysis of 16 variable-number tandem-repeat (VNTR) loci, as previously described [55]. For each sample, four multiplex PCR reactions were performed that generated 16 amplicons and allele copy number was determined by denaturation of amplicons and capillary gel electrophoresis on the sequencer ABI 3130 Genetic Analyzer, using the internal molecular weight sizing standards LIZ 500 (Thermo Fisher Scientific, MA, USA) and copy number definition with the Peak Scanner software (Applied Biosystems, MA, USA).

### Statistical analysis

All numerical data were analyzed using nonparametric tests, with Dunn’s multiple comparisons test to compare relevant groups, or Mann Whitney test to compare continuous variables. All statistical analyses were performed with GraphPad Prism software.

### Ethics statement

The use of rabbits in this study was supported by the authorization granted by the Committee on Ethics in the Use of Animals of UFRRJ (CEUA-UFRRJ) (Rio de Janeiro, Brazil) under No. 380/2013, process 23083.006255 / 2013–25. The use of mice was approved in license number 219/11 by the Animal Welfare Committee of Sagrado Coração University (CEUA-USC) (São Paulo, Brazil), responsible for animal care and inspections at the Instituto Lauro de Souza Lima where all mouse experiments were performed. All procedures were performed in accordance with the Brazilian guide for the production, maintenance and use of animals in teaching or scientific research activities from CONCEA (Conselho Nacional de Controle de Experimentação Animal). In the present work we used the *M*. *leprae* strain PA4 isolated from a skin lesion of an anonymous relapsed multibacillary patient from Igarapé Açú, Pará, Brazil, maintained in nude mice in the *M*. *leprae* Biobank at Lauro de Souza Lima since 2008. The Lauro de Souza Lima Research Ethics Committee (ILSL-CEP) was notified and permitted the use of this sample.

## Results and discussion

The main requirement of a good arthropod vector is to be able to keep a pathogen viable in its tissues until the next blood meal, and to transmit it alive to the bloodstream or tissues of the host during feeding. Some tick-borne pathogens such as *Rickettsia parkeri* are capable of infecting the ovary of the vector arthropod [56], thereby spreading to the offspring which, in the case of the tick *A*. *sculptum*, comprise between 5000 and 8000 eggs per female [57].

**Fig 1:**
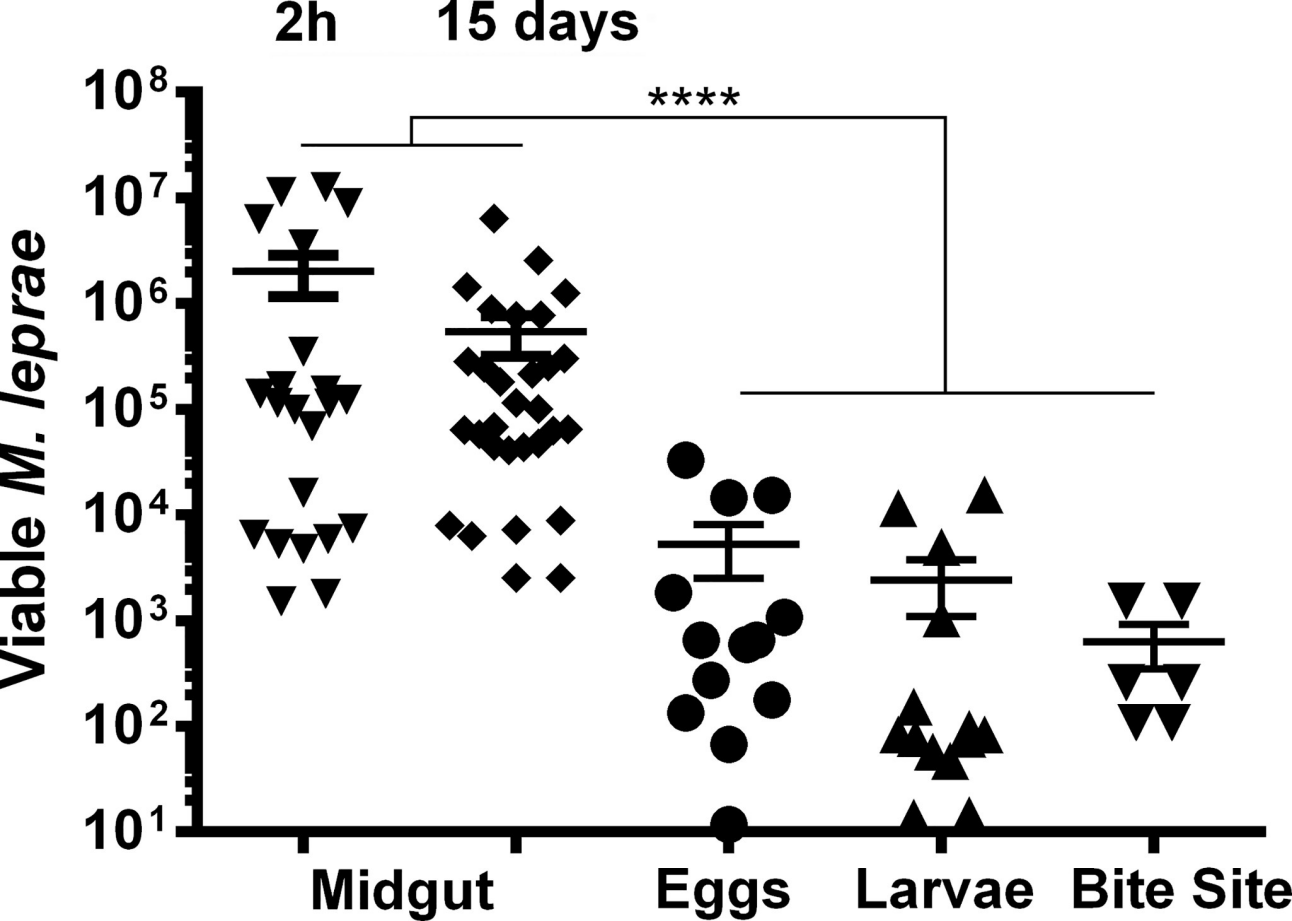
Viability of *Mycobacterium leprae* in different tick tissues after artificial infection of nearly-engorged female *Amblyomma sculptum*. Analysis of viability in *A*. *sculptum* midgut 2 hours (2h) and 15 days after artificial feeding, in eggs and larvae, and in skin biopsies from larval feeding sites. Each data point represents an engorged female, a pool of eggs or larvae, or a rabbit skin biopsy fragment. Levels of 16S rRNA (viable bacillus marker) and 16S rDNA (*M*. *leprae* genome normalizer) were determined by qPCR. Through generation of qPCR standard curves by titration of known numbers of live *M*. *leprae* bacilli in different tissues, values were converted into number of genomes (viable *M*. *leprae*, y-axis). Combined results from three independent experiments, in which none of the negative control samples showed detectable signal; **** indicates p <0.0001 by Dunn’s multiple comparisons test.

Since *M*. *leprae* is only cultivable *in vivo*, rapid quantification of its viability in a non-sterile sample such as tick midgut is a challenge. In order to test the ability of *A*. *sculptum* to sustain *M*. *leprae* infection, we fed nearly-engorged adult female ticks with rabbit blood containing 10^7^ live *M*. *leprae*/ml. This concentration was chosen to mimic heavily-infected armadillo blood, as reticuloendothelial tissue of these animals can contain more than 10^9^ *M*. *leprae*/g [58], and was considered necessary to perform a reliable quantification of live *M*. *leprae* in the different samples. The amount of viable bacteria was determined in tick midguts at 2 h and 15 days by the method of Martinez and collaborators [44], in which the relationship between the number of copies of the 16S ribosomal RNA and the DNA coding for this bacterial gene is measured by qPCR (Fig 1). As PCR efficiency may vary between the various tissue samples due to their intrinsic differences, we performed titration curves of living bacilli for all analyzed materials, identifying the 16S rRNA:16S rDNA ratio corresponding to the number of viable *M*. *leprae* genomes in each tissue type. The 16S rRNA gene was used to test viability because it is the most sensitive and reliable target, due to its abundance and its being completely degraded 48h after *M*. *leprae* inactivation [59].

In our artificial feeding model, each tick would theoretically be able to receive ~2x10^6^ bacilli, since they usually ingested approximately 200 μL of blood per feed, although wide variation was observed in the amount of live *M*. *leprae* per tick midgut at 2 h post infection (Fig 1). This variation in *M*. *leprae* presence, reflecting differences in blood intake, was similarly seen in subsequent samples of eggs and larvae. Nevertheless, we showed that engorged female *A*. *sculptum* were able to maintain the viability of *M*. *leprae* in their midguts for at least 15 days after the blood meal, a time point equivalent to about half-way through the oviposition period [60].

At the second day after the blood meal, the artificially-fed females started to lay eggs. We demonstrated that many of the eggs were infected, by detection of *M*. *leprae* 16S rRNA in this material, as well as in larvae that hatched from eggs after 30 days of incubation at 28°C in a humid chamber (Fig 1). More importantly, we allowed some of these larvae to feed on rabbits. After five days of exposure, the tick attachment sites were identified and biopsied. Analysis of this material also identified over 10^3^ live *M*. *leprae* in some of the bite sites (Fig 1), suggesting that during blood feeding the larvae could have inoculated an amount of *M*. *leprae* sufficient to infect a susceptible armadillo [61].

**Fig 2:**
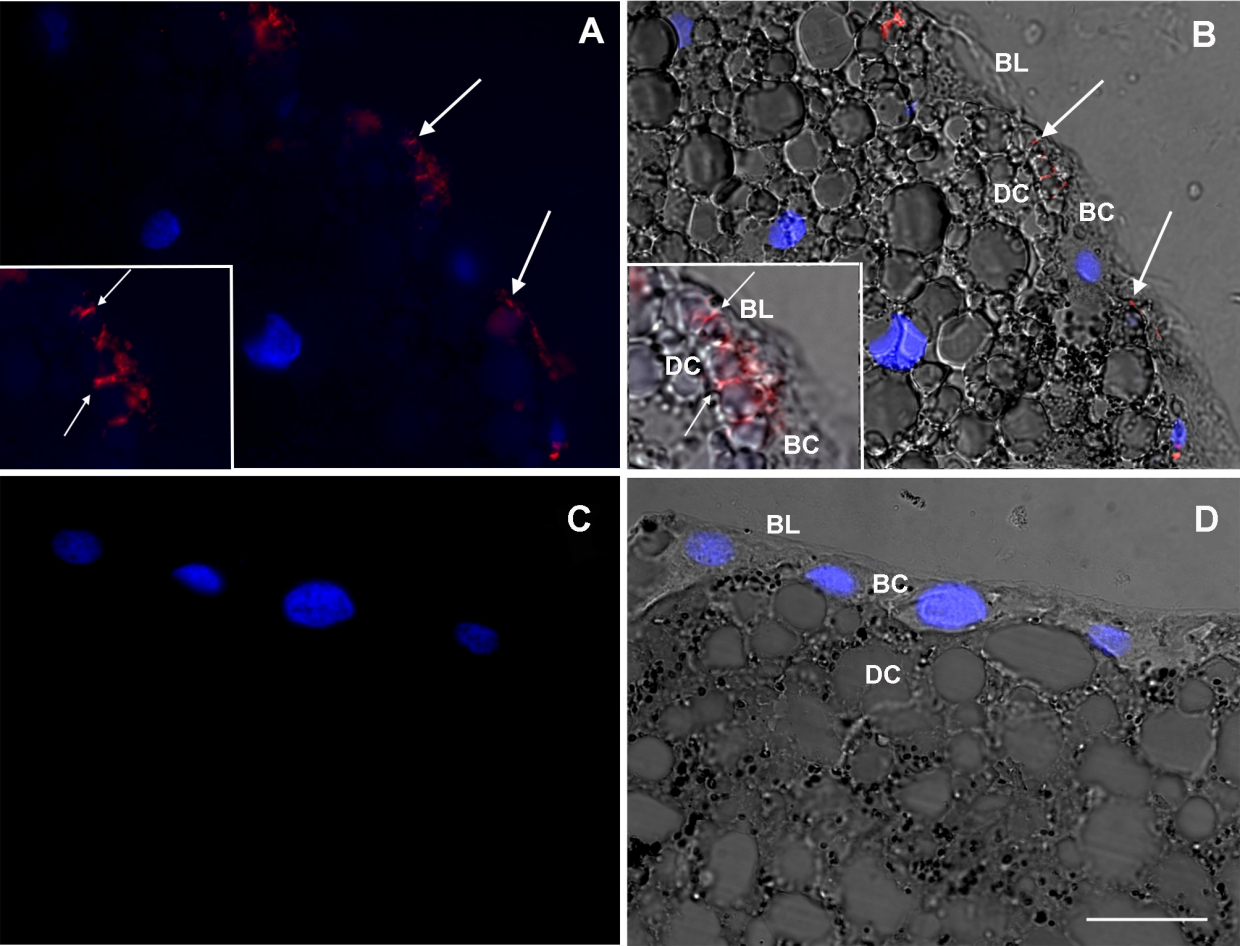
Immunolocalization of *Mycobacterium* LAM cell wall antigen in *Amblyomma sculptum* midgut 15 days after infection. Midguts from *A*. *sculptum* females after artificial feeding with uninfected (C, D) or *M*. *leprae*-infected (A, B) rabbit blood were fixed and sectioned. Panels A and C were generated through the merging of red channel image (anti-LAM + anti-mouse Alexa 594) with blue channel image (DAPI nuclear staining). Panels B and D were generated by merging with the differential interference contrast images. Almost all mycobacteria were observed in the basal region of the digestive cells (DC) (arrows). The images are representative of thirty fields, generated from three independent experiments: BL = basal lamina; BC = basophilic cell. Scale bar represents 15μm in main images and 7.5μm in insets.

Direct visualization of mycobacteria which we believed to be *M*. *leprae* in the various tissues was obtained through immunolocalization of LAM, a glycolipid exclusive to the genus *Mycobacterium*. At 15 days post feeding, when digestion of the blood meal would have been completed by the majority of the individual ticks [62], bacilli were visible in the basal region of the digestive cells (Fig 2), at a site where these cells are inserted between the midgut epithelial basophilic cells and make contact with the basal lamina, thereby gaining direct access to the hemocoel [63].

**Fig 3:**
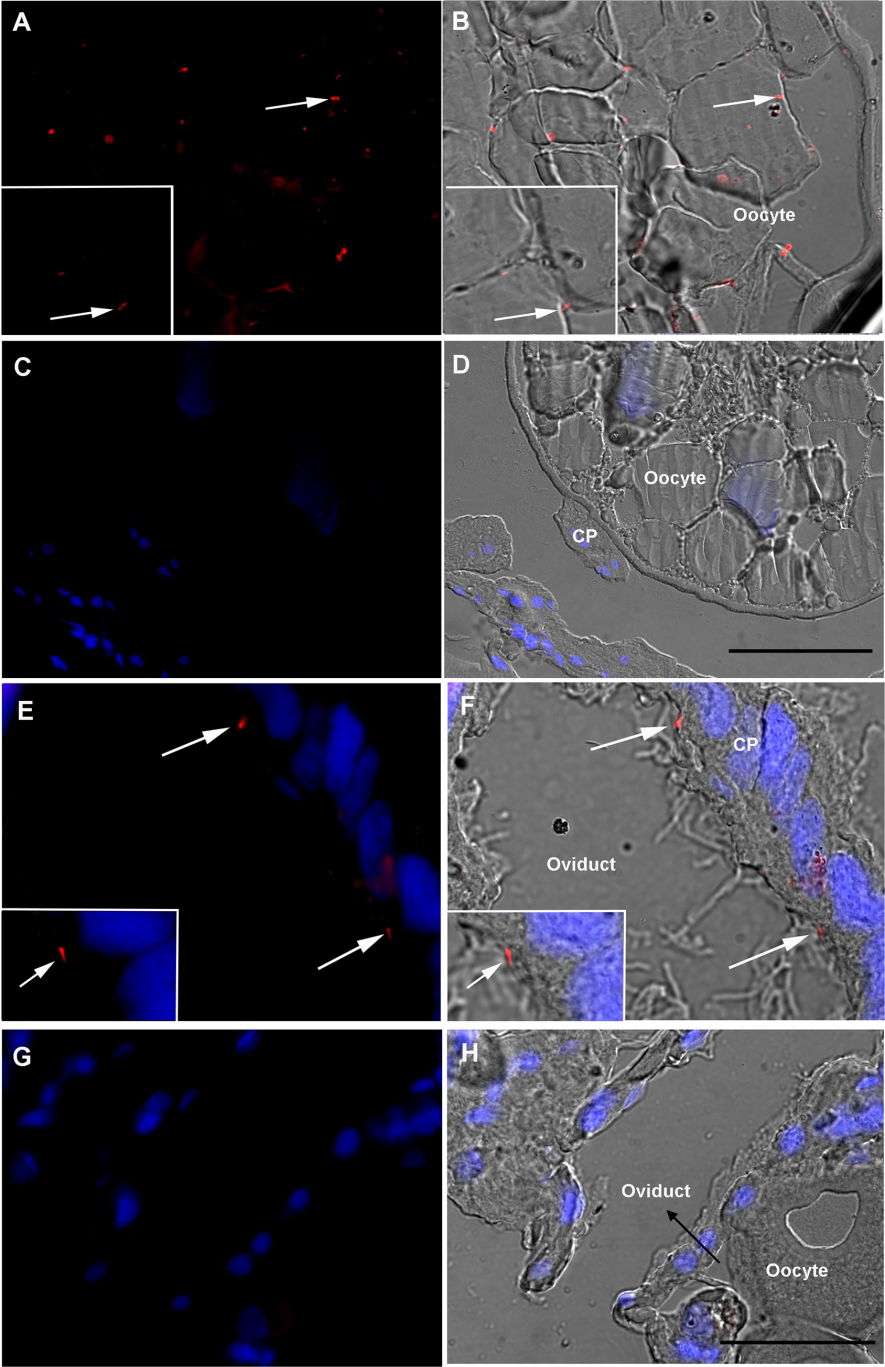
Immunolocalization of *Mycobacterium* LAM cell wall antigen in *Amblyomma sculptum* ovary 2 days after infection. Ovaries from *A*. *sculptum* females after artificial feeding with uninfected (C, D, G, H) or *M*. *leprae*-infected (A, B, E, F) rabbit blood were fixed and sectioned. Panels A, C, E, and G were generated through the merging of red channel image (anti-LAM + anti-mouse Alexa 594) with blue channel image (DAPI nuclear staining). Panels B, D, F and H were generated by merging with the differential interference contrast images. Bacilli (arrows) were seen in oocytes (A-B) and pedicels (E-F). The images are representative of thirty fields, generated from three independent experiments; CP = pedicel. Scale bar represents 50μm in main images and 25μm in insets.

**Fig 4:**
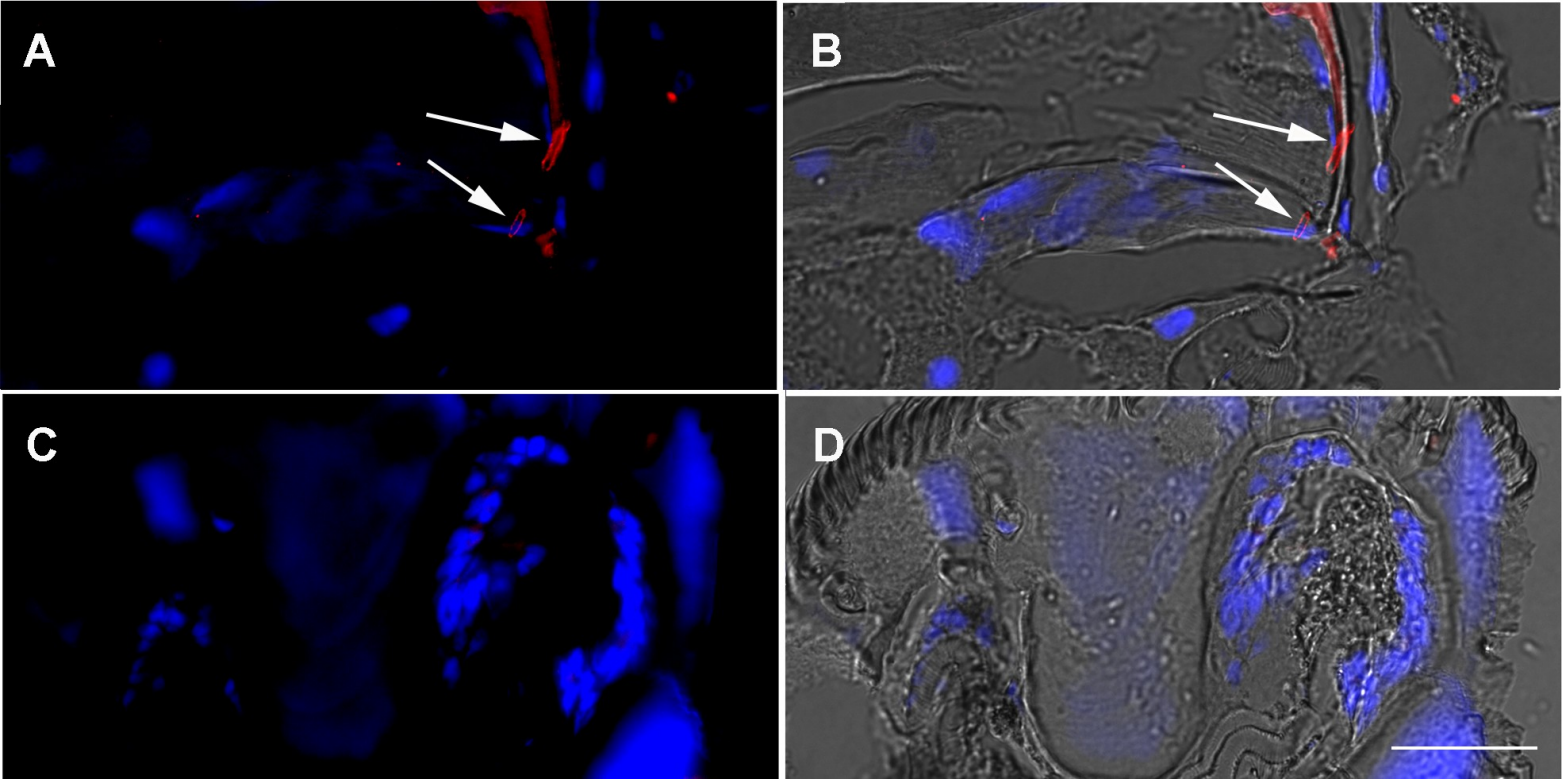
Immunolocalization of *Mycobacterium* LAM cell wall antigen in *Amblyomma sculptum* larvae. Larvae that hatched from eggs laid by *A*. *sculptum* females after artificial feeding with uninfected (C, D) or *M*. *leprae*-infected (A, B) rabbit blood were fixed and sectioned. Panels A and C were generated through merging red channel image (anti-LAM + anti-mouse Alexa 594) with blue channel image (DAPI nuclear staining). Panels B and D were generated by merging with differential interference contrast images. Small numbers of mycobacteria (arrows) can be seen inside the digestive tract of larvae derived from infected females. Images representative of nineteen larvae observed in three independent biological replicates. Scale bar represents 15μm.

**Fig 5:**
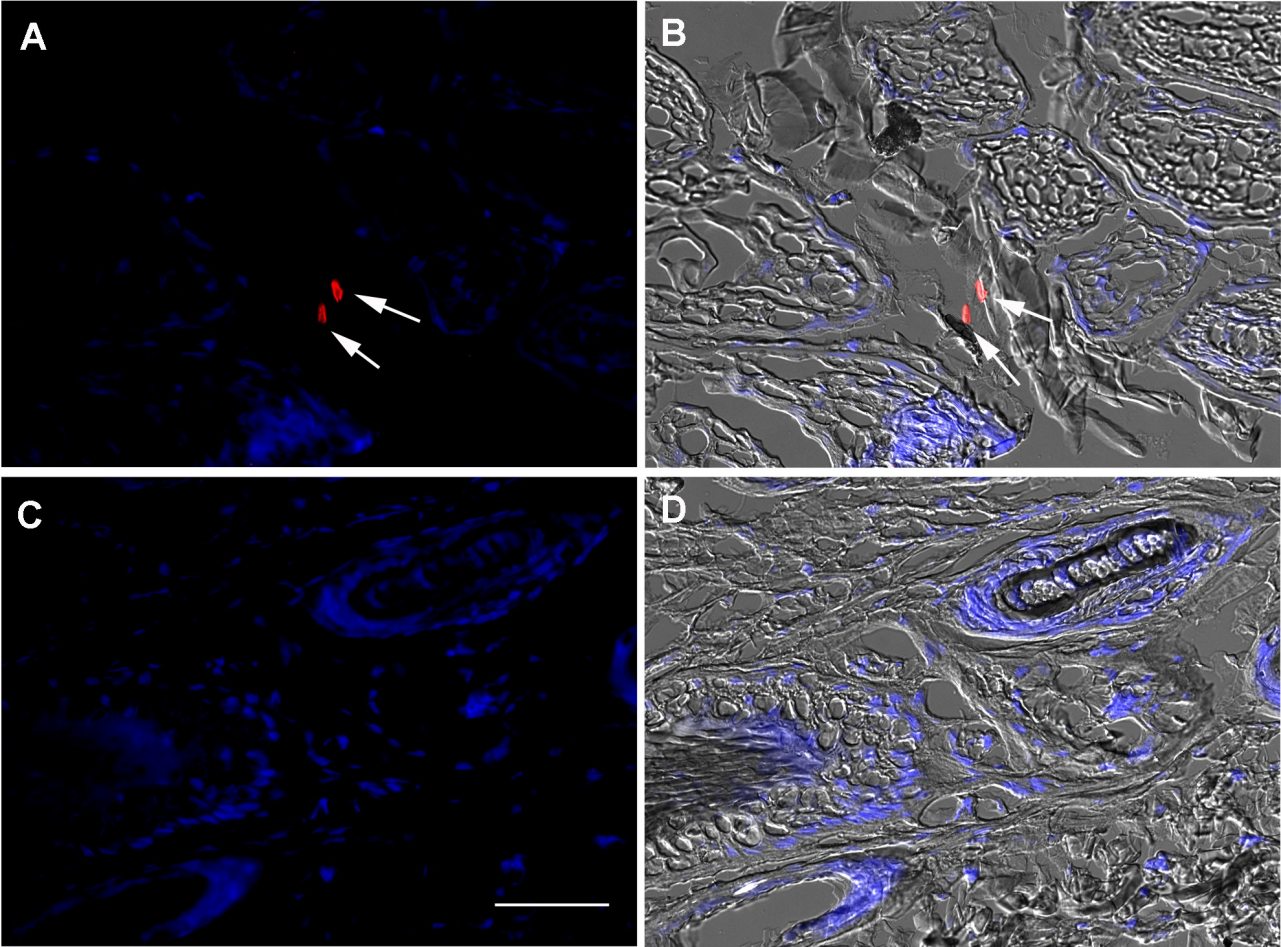
Immunolocalization of *Mycobacterium* LAM cell wall antigen in skin biopsies from a rabbit infested with *Amblyomma sculptum* larvae. Biopsies of bite sites collected from rabbits following 5 days’ infestation with larvae hatched from eggs laid by *A*. *sculptum* females after artificial feeding with uninfected (C, D) or *M*. *leprae*-infected (A, B) rabbit blood were fixed and sectioned. Panels A and C were generated through the merging of red channel image (anti-LAM + anti-mouse Alexa 594) with blue channel image (DAPI nuclear staining). Panels B and D were generated by merging with differential interference contrast images. In panels A and B, LAM-positive bacilli (arrows) can be seen in the area where the larval hypostome penetrated the rabbit skin. These images are representative of four biopsies obtained from two independent experiments. Scale bar represents 40μm.

Demonstration of escape of *M*. *leprae* from midgut to hemocoel was provided by LAM immunolocalization revealing the presence of intact bacilli in both oocytes and the pedicels that attach the oocytes to the oviduct in infected females 2 days after feeding (Fig 3), as well as in the digestive tract of larvae that hatched from these eggs (Fig 4), suggesting vertical transmission of *M*. *leprae*, a phenomenon also reported for other tick-borne pathogens such as *Rickettsia rickettsii* [28]. This was further supported by the presence of mycobacteria at the larval bite sites, shown by immunolocalization of LAM in the rabbit skin biopsies (Fig 5). We did not observe any difference in the number of inflammatory cells infiltrating the host tissues, when comparing infected and control skin biopsies. The bacilli were observed mostly around the lesions generated by the tick mouthparts. The absence of any LAM signal in the tick and rabbit control samples (Figs 2–5) makes it very unlikely that the LAM-positive cells found in the tissues from infected samples might be attributed to another *Mycobacterium* species, since the ticks and rabbits in both groups (infected and control) came from the same colony and animal facility respectively.

**Fig 6:**
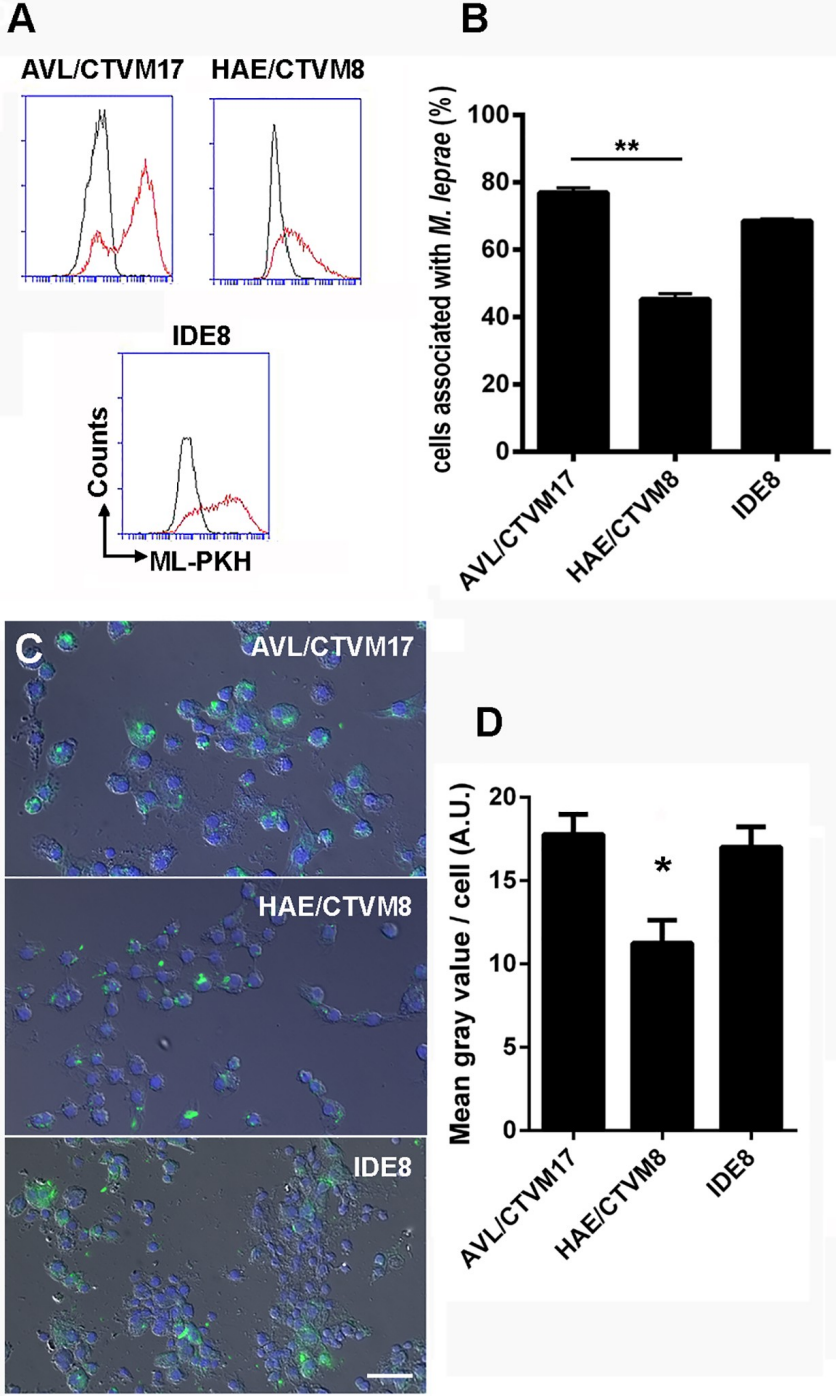
*Mycobacterium leprae* is able to associate with different tick cell lines. A) Histograms generated by flow cytometry showing association between *M*. *leprae* labelled with PKH67 and three tick cell lines: AVL/CTVM17, HAE/CTVM8 and IDE8. Gray lines represent uninfected tick cells and red lines represent cells associated with the fluorescent bacilli. B) Quantification of the histograms in (A) demonstrating a high level of association between the pathogen and cell lines AVL/CTVM17 and IDE8. C) Fluorescence microscopy 24h after infection of tick cell lines with *M*. *leprae* labelled with the green fluorophore PKH67. D) Quantification of the mean values of fluorescence intensity/cell in (C), for which sixty photos were analyzed from three independent experiments. Scale bar represents 20μm; ** = p <0.005 and * = P <0.05 by Dunn’s multiple comparisons test.

The conclusion that *A*. *sculptum* ticks are potential leprosy vectors led us to investigate whether *M*. *leprae* could infect embryonic or larval cells from different tick species *in vitro*. We used three different tick cell lines: AVL/CTVM17 (*A*. *variegatum*), HAE/CTVM8 (*H*. *anatolicum*) and IDE8 (*I*. *scapularis*), all of which are derived from human-biting tick species [64]. *A*. *variegatum* was chosen because there were no cell lines available from *A*. *sculptum*, *H*. *anatolicum* is a tick species widely distributed in India where leprosy is endemic, and the *I*. *scapularis* cell line IDE8 is known to be highly permissive to infection with many different bacterial species [30]. Tick cell cultures were inoculated with *M*. *leprae* labeled with the green fluorophore PKH67. We observed by flow cytometry (Fig 6A and 6B) and by microscopy (Fig 6C and 6D) that *M*. *leprae* was capable of associating with all three cell lines at rates between 45.3% (HAE/CTVM8) and 76.9% (AVL/CTVM17) (Fig 6B). The levels of *M*. *leprae-*cell association determined by quantification of fluorescence/cell through microscopy corroborated the flow cytometry analysis, confirming that AVL/CTVM17 and IDE8 cells were more susceptible to *M*. *leprae* attachment than HAE/CTVM8 cells (Fig 6D).

**Fig 7:**
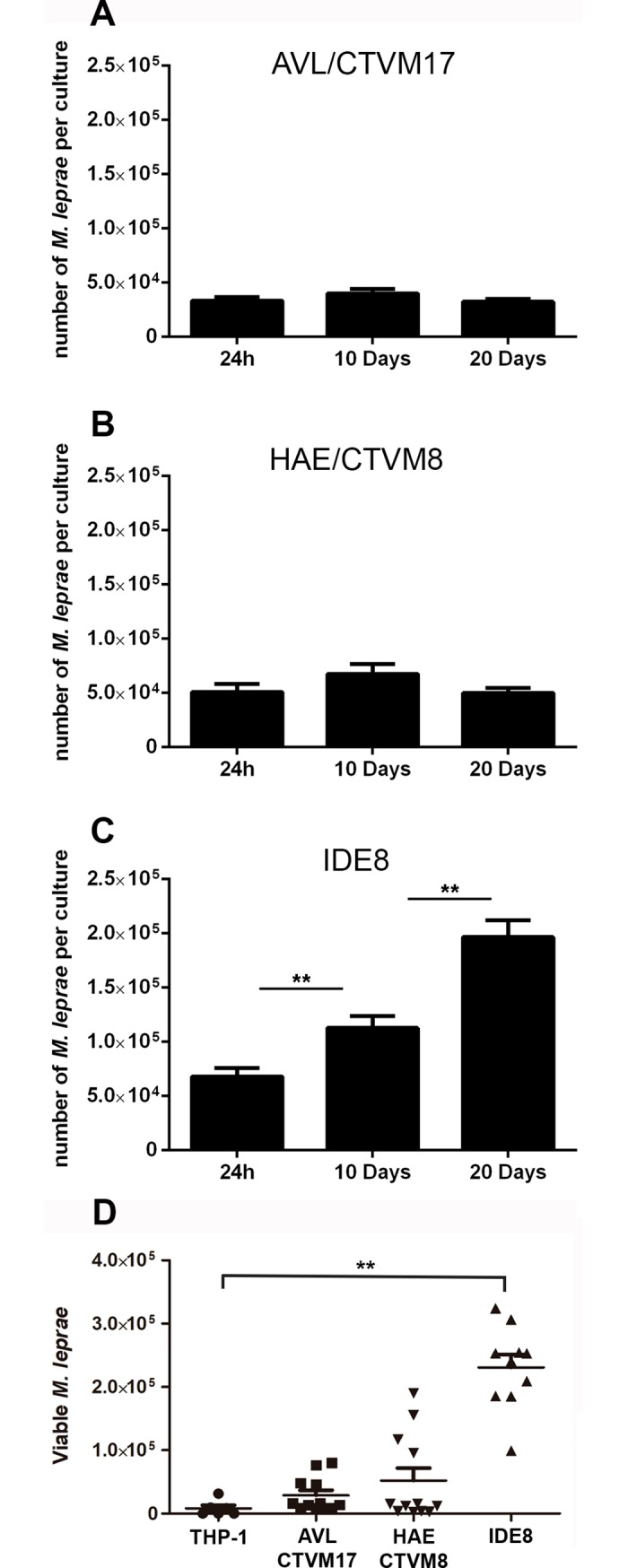
*Mycobacterium leprae* is able to grow in tick cell cultures. Tick cell lines AVL/CTVM17, HAE/CTVM8 and IDE8 were infected with *M*. *leprae*; the initial inoculum (24h) and the bacillary load after 10 and 20 days of incubation at 30°C were determined by cell lysis and counting of total acid-fast bacilli. In (A) AVL/CTVM17 and (B) HAE/CTVM8 cells there was a slight, non-significant increase in bacteria over the first 10 days followed by a decrease, whereas in (C) IDE8 cells there was a significant increase in bacterial numbers over the 20-day period. Each bar represents the mean of nine independent cultures performed using three different *M*. *leprae* preparations. (D) Viable *M*. *leprae* titer in human monocyte THP-1 cells and tick cell lines after 20 days of infection. Levels of 16S rRNA (viable bacillus marker) and 16S rDNA (*M*. *leprae* genome normalizer) were determined by qPCR. Through generation of qPCR standard curves by titration of known numbers of live *M*. *leprae* bacilli in different cell cultures, values were converted into number of genomes (viable *M*. *leprae*, y-axis). Combined results from three independent experiments, in which none of the negative control samples showed detectable signal; ** = p <0.0001 by Dunn’s multiple comparisons test.

We then investigated whether *M*. *leprae* would be able to remain viable within these cells, grown at 30°C. For this, we inoculated the three tick cell lines with *M*. *leprae* and the total numbers of acid fast bacilli in the cultures were determined 2 h, 10 days and 20 days after infection (Fig 7). Due to their thick cell wall made up of mycolic acids, dead bacilli are not digested, and can remain within the culture seemingly intact for weeks. Thus, the observed levels of *M*. *leprae* at 20 days in the AVL/CTVM17 and HAE/CTVM8 cultures were possibly due to inactivated or dead bacteria, although these levels were higher than those seen in human THP-1 cells at the same time point (Fig 7D). In contrast, the IDE8 cell line was able to support a detectable increase in the number of bacilli over the 20 day period, showing an *in vitro* doubling time of approximately 12 days, similar to that observed *in vivo* in mouse foot-pads [35]. Although it was previously demonstrated that *M*. *leprae* was able to persist for up to 8 months inside different amoeba species [65,66], this is the first report of a cell culture system in which an actual increase in the number of *M*. *leprae* bacilli was detected, suggesting that replication had occurred.

After 20 days cultivation in the IDE8 cell line, we harvested the bacilli and used them to infect mouse foot pads following the Shepard model [67]. After six months, we confirmed that *M*. *leprae* grown in IDE8 cells were subsequently able to grow inside mouse foot pads in a manner similar to bacilli freshly harvested from nude mice, demonstrating the ability of this tick cell line to maintain *M*. *leprae* viability and infectivity (Table 1). We are currently developing a protocol for continuous *in vitro* cultivation of *M*. *leprae* in IDE8 cells involving addition of fresh uninfected tick cells to existing infected cultures, and/or passaging aliquots of infected cultures onto new uninfected tick cell cultures, as used successfully for other obligate intracellular bacteria [29, 30, 31, 33, 47].

**Table 1:** Viability of tick cell-derived *Mycobacterium leprae* in the Shepard model.

Inoculum	Mouse number				
	1	2	3	4	5
Mice receiving *M*. *leprae* from IDE8 cells	2.5x10^5^*	8.9x10^5^	6.1x10^4^	8.8x10^4^	5.5x10^5^
Mice receiving *M*. *leprae* from IDE8 cells followed by rifampicin treatment	0	0	0	0	0
Mice receiving *M*. *leprae* from mouse footpads	4.0x10^5^	1.3x10^5^	3.5x10^5^	1.8x10^5^	2.5x10^5^

*Number of bacilli recovered from BalbC mouse footpads six months after inoculation with 10^4^ bacteria harvested from IDE8 cells alone (test group) or with subsequent treatment with rifampicin (negative control group), or inoculation with 10^4^ bacteria freshly harvested from mouse footpads (positive control group).

Once we were able, for the first time, to cultivate *M*. *leprae in vitro*, this allowed us to select for antibiotic resistance and transform a *M*. *leprae* strain isolated from a multibacillary leprosy patient using the plasmid pCHERRY3 encoding mCherry fluorescent protein and hygromycin resistance [68]. The efficiency of transformation was about 12 ± 6% (2x10^7^ transformed bacteria per μg plasmid DNA), lower than that observed in *Mycobacterium tuberculosis* using the same plasmid, with which 100% of transformed cells expressed mCherry (54), demonstrating that the *M*. *smegmatis* smyc promoter is not as efficient in *M*. *leprae*. We are now editing the pCHERRY3 plasmid, to replace the smyc promoter with *M*. *leprae* promoters. We believe that this approach will increase our understanding of *M*. *leprae* expression mechanisms and homeostasis.

**Fig 8:**
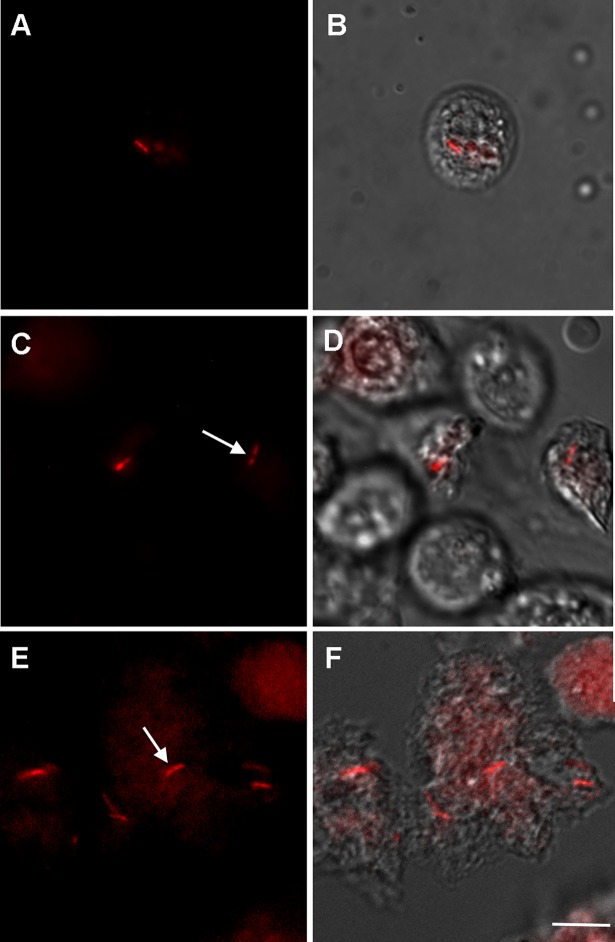
Fluorescent *Mycobacterium leprae* generated by transfection with the plasmid pCHERRY3 within IDE8 tick cells. Fluorescence of the bacteria expressing mCherry was seen after 1 week (A, B), 2 weeks (C, D) and 2 months (E, F) after transformation; at all time points, the majority of fluorescent *M*. *leprae*-mCherry were in division (arrows). Panels A, C and E were generated by conventional fluorescence microscopy, using a 590 nm LED with a Zeiss fluorescence filter 61; panels B, D and F were generated by merging with differential interference contrast images. Scale bar represents 10 μm.

The transformed bacteria, designated *M*. *leprae*-mCherry, demonstrated their ability to grow inside IDE8 cells, as we could see by frequent visualization of transgenic bacteria dividing into two daughter cells at different time points (Fig 8, arrows). There was no difference between the growth rate of electroporated and control bacilli over a 20 day period, and bacterial viability determined weekly by LIVE/DEAD staining ranged between 70 and 90% over 59 days *in vitro*. After two monthsIDE8 cells in the infected culture started to present apparent cytopathic effects, although intracellular *M*. *leprae*-mCherry maintained their ability to express mCherry (Fig 8).

At this point we developed a real-time viability analysis of *M*. *leprae*, by exposure of IDE8 cells infected with *M*. *leprae*-mCherry IDE8 cells to ofloxacin. After 5 h of incubation, time-lapse video microscopy showed, for the first time, real-time *M*. *leprae* inactivation by an antibiotic, determining precisely the timing of cell wall permeabilization and leak of the mCherry fluorescent probe, a process which started 6 h after exposure to the antibiotic and took approximately 1 h (Supporting Video 1). The mycobacteria cultured in IDE8 cells (Fig 7) and subsequently inoculated into BalbC mice (Table 1) or transformed by the pCHERRY3 plasmid (Fig 8), were positively identified as *M*. *leprae* by VNTR sequencing (Table 2). Cultivable contaminating microorganisms were not observed among any of these samples 72h after inoculation of blood agar plates, and uninfected IDE8 cells did not exhibit any fluorescence.

**Table 2:** VNTR genotypes of *M*. *leprae* samples analyzed before (original samples) and after cultivation in the tick cell line IDE8 (Thai-53 strain) and *in vitro* transformation (PA84 strain).

Samples	6–3	AT17	GGT5	GTA9	AC8B	AC8A	AT15	21–3	TTC	6–7	27–5	23–3	12–5	18–8
THAI-53*	3	10	5	9	7	11	13	3	14	6	5	2	4	8
THAI-53 after 20 days’ growth in IDE8	3	10	5	9	ND	11	ND	3	ND	6	5	2	ND	8
PA84*	3	14	4	10	7	ND	17	2	11	6	5	2	4	3
PA84 *M*. *leprae* -mCherry	3	14	4	10	7	ND	17	2	11	6	5	2	4	3

*Original samples.

ND: unable to characterize due to negative PCR.

The distribution and prevalence of leprosy in different countries of the world challenges the hypothesis pointing to untreated multibacillary patients as being the only source of contagion, since countries that receive a large flow of immigrants from hyperendemic areas, such as the United Kingdom and USA, do not register high numbers of autochthonous cases of the disease [3]. Moreover, in hyperendemic countries such as Brazil, most of the new cases are not related to large agglomerations of poor citizens, such as are found in the periphery of large urban centers where the transmission of respiratory infections such as tuberculosis is facilitated. In Brazil leprosy is actually concentrated at the agricultural frontier [69]. In fact, a study carried out in the Brazilian Amazon successfully correlated detection rates of new cases of Hansen's disease with deforestation activity [9]. Armadillos are recognized as a possible source of *M*. *leprae* infection in the southern USA and northern Brazil, where the disease is considered by some authors to be a zoonosis [11, 10]. Autochthonous US leprosy patients live in areas where infected armadillos can be found, and direct contact with these animals in Brazil and USA is also associated with a higher risk of being infected [7, 10, 11, 70]. We hypothesized that the numbers of new cases of leprosy recorded annually may be related to person-to-person transmission plus infection via ingestion of, and/or contact with, *M*. *leprae-*infected wild animals such as armadillos [11], squirrels [14], monkeys [13,71] and hematophagous arthropods that feed on these animals such as kissing bugs [22] and ticks.

Ticks have a high potential to be vectors because they are long-lived and may take their blood meal from multiple animal species during their life cycle, plus the fact that many of the microorganisms that they harbor can be transmitted vertically from the mother tick to her progeny via the eggs. For these reasons, ticks are responsible for transmitting more species of pathogen to humans and domestic animals than any other arthropod. The IDE8 cell line, used successfully in this study to propagate *M*. *leprae in vitro*, was isolated from embryos of the blacklegged tick *I*. *scapularis*. Ticks (Acari: Ixodidae) have been reported in the literature to parasitize armadillos in several regions of the Americas [26,72–74]. Their role in the transmission of leprosy between armadillos, and from armadillos to humans, must be investigated. In the near future we intend to start the search for ticks naturally infected with *M*. *leprae*, in order to best understand the putative role of ticks in leprosy transmission among squirrels in the UK [14] and armadillos in the Americas [75–78].

Throughout the last century, the search for a laboratory animal model able to support growth of *M*. *leprae* involved several species, but significant progress was only made in 1960, when Charles Shepard for the first time described local and limited growth of the pathogen in the mouse foot pad, mainly based on the low temperature of this environment [53]. This method evolved into the use of the athymic nude mouse Crl:NU(NCr)-Foxn1^nu^ for the cultivation of *M*. *leprae* [79]. Another protocol to produce *M*. *leprae* involves intravenous inoculation of *Dasypus novemcinctus* armadillos. The low body temperature of this animal (30–35°C) is also believed to be related to the success of this model in generating high bacillary numbers in internal organs [80]. Until now only animal models have been able to produce *M*. *leprae* with sufficiently high numbers and viability for experimental use [35,39].

On the other hand, many attempts have been made in the past to cultivate *M*. *leprae* axenically in different kinds of media. Recently, Amako and colleagues made some progress with a modified Kirchner Medium, replacing bovine serum with human plasma supplemented with egg-yolk extract, pyruvate and transferrin [81]. Although the authors successfully observed *M*. *leprae* genome duplication using droplet digital PCR, *M*. *leprae* growth using this axenic protocol appeared to be abnormally slow, with a doubling time close to 70 days [81]. In contrast, using IDE8 cells we observed an apparent doubling time of approximately 12 days, much closer to that observed *in vivo* (Fig 7C).

In a parallel to the revolution that occurred in research on bovine anaplasmosis in the 1990s, when the obligate intracellular bacterium *Anaplasma marginale* was first cultivated in tick cell lines [33], the *in vitro* production of *M*. *leprae* in the tick cell line IDE8 has potential for enormous impact in studies on the biology of the pathogen, large-scale production of its antigens and screening of new drugs. Production of *M*. *leprae* in cell bioreactors becomes a distinct possibility, generating relatively pure and LPS-free bacilli, which is difficult to achieve using nude mouse foot pads or armadillo liver and spleen. Our findings will facilitate site-directed mutagenesis studies in *M*. *leprae*, to determine the impact of genes of interest on the physiology of the bacillus. In addition, we now have the means and potential to understand the role of the abundant noncoding regions of the *M*. *leprae* genome, finally unravelling the secrets of one of the most enigmatic pathogens that still afflict humankind.

## Supporting information

S1 Video Visualization of antibiotic-mediated killing of intracellular *Mycobacterium leprae*. Time-lapse microscopy shows the exact moment of death of an *M*. *leprae*-mCherry bacterium in an IDE8 cell commencing 5 h after medium supplementation with 150 μg / mL ofloxacin. Scale bar represents 10 μm.
(AVI)
